# A decade of human metapneumovirus in hospitalized children with acute respiratory infection: molecular epidemiology in central Vietnam, 2007–2017

**DOI:** 10.1038/s41598-023-42692-z

**Published:** 2023-09-21

**Authors:** Hirono Otomaru, Hien Anh Thi Nguyen, Hien Minh Vo, Michiko Toizumi, Minh Nhat Le, Katsumi Mizuta, Hiroyuki Moriuchi, Minh Xuan Bui, Duc Anh Dang, Lay-Myint Yoshida

**Affiliations:** 1 Department of Pediatric Infectious Diseases, Institute of Tropical Medicine, Nagasaki University, Nagasaki, Japan; 2 Department of Bacteriology, National Institute of Hygiene and Epidemiology, Hanoi, Vietnam; 3 Department of Pediatrics, Khanh Hoa General Hospital, Nha Trang, Vietnam; 4 Antimicrobial Resistance Research Centre, National Institute of Infectious Disease (NIID), Sinjuku, Japan; 5 Tay Nguyen Institute of Science Research, Vietnam Academy of Science and Technology (VAST), Da Lat, Vietnam; 6 Yamagata Prefectural Institute of Public Health, Yamagata, Japan; 7 Department of Pediatrics, Nagasaki University Graduate School of Biomedical Sciences, Nagasaki, Japan; 8 Khanh Hoa Health Service Department, Nha Trang, Vietnam; 9 National Institute of Hygiene and Epidemiology, Hanoi, Vietnam; 10 Graduate School of Biomedical Sciences, Nagasaki University, Nagasaki, Japan

**Keywords:** Infectious diseases, Molecular evolution

## Abstract

Human metapneumovirus (hMPV) can cause severe acute respiratory infection (ARI). We aimed to clarify the clinical and molecular epidemiological features of hMPV. We conducted an ARI surveillance targeting hospitalized children aged 1 month to 14 years in Nha Trang, Vietnam. Nasopharyngeal swabs were tested for respiratory viruses with PCR. We described the clinical characteristics of hMPV patients in comparison with those with respiratory syncytial virus (RSV) and those with neither RSV nor hMPV, and among different hMPV genotypes. Among 8822 patients, 278 (3.2%) were hMPV positive, with a median age of 21.0 months (interquartile range: 12.7–32.5). Among single virus-positive patients, hMPV cases were older than patients with RSV (*p* < 0.001) and without RSV (*p* = 0.003). The proportions of clinical pneumonia and wheezing in hMPV patients resembled those in RSV patients but were higher than in non-RSV non-hMPV patients. Seventy percent (n = 195) were genotyped (A2b: n = 40, 20.5%; A2c: n = 99, 50.8%; B1: n = 37, 19%; and B2: n = 19, 9.7%). The wheezing frequency was higher in A2b patients (76.7%) than in those with other genotypes (*p* = 0.033). In conclusion, we found a moderate variation in clinical features among hMPV patients with various genotypes. No seasonality was observed, and the multiple genotype co-circulation was evident.

## Introduction

Human metapneumovirus (hMPV), a member of the *Pneumoviridae* family, was first discovered in 2001 as a causative agent of acute respiratory infection (ARI)^1^. A study with meta-analysis reported that a hMPV prevalence in hospitalized ARI patients was estimated to be 6.24% (95% confidence interval: 5.25–7.30)^2^ with a significant heterogeneity worldwide^3^. HMPV seropositivity is reported to exceed 90% by 2 years old^4^. Previous studies have shown elevated neutralizing serum antibody titers with infection in both children^5^ and adults^6^; however, antibodies are not maintained at sufficiently high levels to prevent reinfection. In children, upper respiratory tract infection symptoms, hypoxia, and wheezing are common. Clinical characteristics of children with hMPV infection have been reported to be indistinguishable from those of children infected with respiratory syncytial virus (RSV), a common respiratory pathogen, another member of the *Pneumoviridae* family. Although children with hMPV tend to be older than those with RSV^7,8^, a significant number of children are hospitalized with severe symptoms of lower respiratory tract infection, such as bronchiolitis and pneumonia^9^. A cohort study in adults has reported that older adults often develop bronchitis and pneumonia^10^, which place an hMPV-associated disease burden on individuals and public health.

Of the two main immunogenic surface proteins of hMPV, attachment glycoprotein (G) and fusion (F) proteins, the F protein has been shown to possess antibody-neutralization epitopes^11,12^. Although neither vaccine nor monoclonal antibody has yet been developed, F protein can induce neutralizing antibodies in vitro and in animal experiments^13–16^, and a human anti-F protein antibody cross-neutralizing RSV and hMPV has been also identified^17^; hence, the F protein is expected to be a promising useful target for the development of vaccines or monoclonal antibody. HMPV is classified into two subgroups, A and B, based on its genetic and antigenic differences, and further classified into genotypes A1, A2, B1, and B2 according to genetic differences^18,19^. HMPV genotype-specific neutralizing antibody titers in children are thought to fluctuate, influenced by the prevalent genotype^5^. Monitoring hMPV variants and determining their evolutionary dynamics are therefore important in devising preventive measures against hMPV.

Long-term surveillance data are yet to be thoroughly analyzed to describe the molecular evolutionary pattern of hMPV. Baseline data of hMPV, such as disease burden, evolutionary characteristics, and clinical features, are necessary to evaluate the impact of therapeutic or preventive strategies on public health. The present study aimed to describe the demographic and clinical characteristics of patients with single detection of hMPV, in comparison with patients with and without RSV infection. We also aimed to elucidate the evolutionary aspects of the hMPV F gene and concurrent changes in clinical and epidemiological features by integrating surveillance data and the genotype of hMPV.

## Results

### Yearly detection of hMPV among hospitalized pediatric patients with ARI

**Figure 1:**
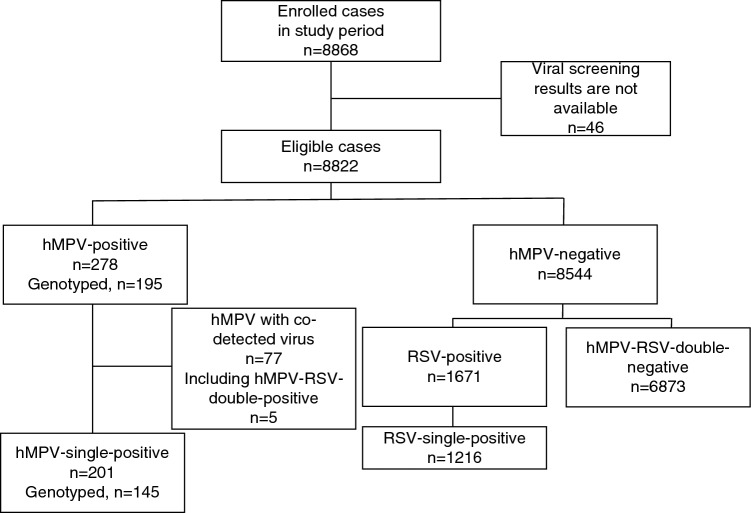
Schematic flowchart of the study participants. *hMPV* human metapneumovirus.

**Figure 2:**
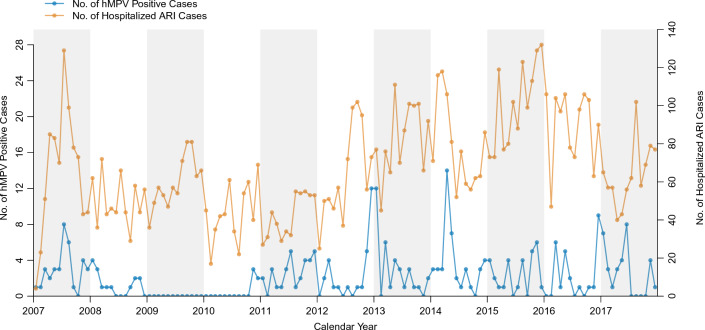
The monthly number of hMPV-positive cases detected through ARI surveillance in central Vietnam, 2007–2017. The numbers of hMPV cases are described using the blue line and the left side of the y-axis. The numbers of ARI cases are described using the orange line and the right side of the y-axis. *hMPV* human metapneumovirus, *ARI* acute respiratory infection.

In total, 8868 children hospitalized with ARI were eligible and enrolled in surveillance during the study period. After excluding 46 children whose viral screening results were unavailable, 8822 children were included in the analysis (Fig. 1). As a result of PCR screening and hemi-nested PCR confirmation, 278 (3.2%) patients were positive for hMPV, including those positive for both hMPV and RSV (n = 5). Annual hMPV-positive cases ranged between 0 and 6.2%, indicating continuous circulation of hMPV, other than during 2009–2010; when only five hMPV-positive cases were detected (Table 1). The monthly number of hMPV cases is summarized in Fig. 2 and Supplementary Fig. 1. These described that there was no remarkable seasonal circulation trend of hMPV infection. In contrast, clear seasonality was shown for RSV infection (Supplementary Fig. 1), although RSV cases were also fewer in 2009 all year round than in other years.

**Table 1:** The yearly number of casess with hMPV during 2007–2017.

Year	No. of eligible cases (n = 8822)	No. of hMPV positive cases (n = 278)	Percent of hMPV positive (95% CI)	No. of eligible cases under five (n = 8207)	No. of hMPV positive cases under five (n = 265)	Percent of hMPV positive cases under five (95% CI)
2007	782	35	4.5 (3.2–6.2)	748	33	4.4 (3.1–6.2)
2008	600	15	2.5 (1.5–4.2)	554	15	2.7 (1.6–4.5)
2009	715	0	0.0 (0.0–0.7)	667	0	0.0 (0.0–0.7)
2010	522	5	1.0 (0.4–2.4)	495	5	1.0 (0.4–2.5)
2011	498	31	6.2 (4.3–8.8)	453	27	6.0 (4.0–8.7)
2012	762	28	3.7 (2.5–5.3)	708	26	3.7 (2.5–5.4)
2013	972	34	3.5 (2.5–4.9)	907	33	3.6 (2.6–5.1)
2014	939	44	4.7 (3.5–6.3)	869	44	5.1 (3.7–6.8)
2015	1197	29	2.4 (1.7–3.5)	1090	26	2.4 (1.6–3.5)
2016	1070	26	2.4 (1.6–3.6)	997	26	2.6 (1.7–3.9)
2017	765	31	4.1 (2.8–5.8)	719	30	4.2 (2.9–6.0)

*hMPV* human metapneumovirus, *CI* confidence interval.

### Genotype distribution

**Figure 3:**
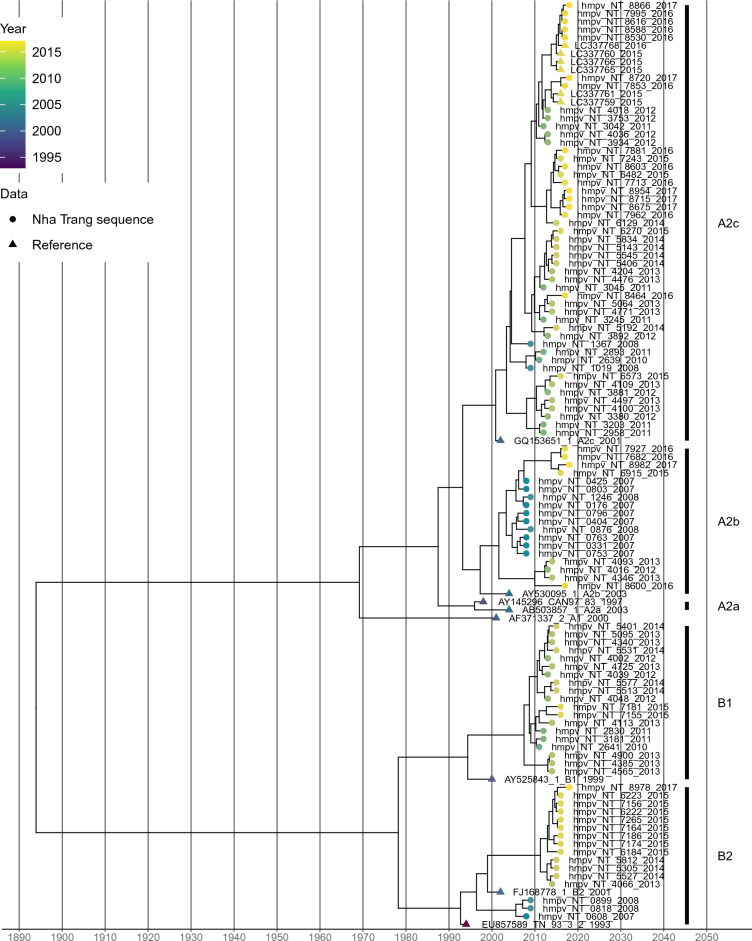
Time-scaled maximum clade credibility tree with the obtained unique hMPV F gene sequences and references. The colors of the terminal tips display the year when cases were enrolled in the study. Tips whose name includes “NT” were obtained in the present study and described as solid circles. Tips described as solid triangles are reference sequences.

**Figure 4:**
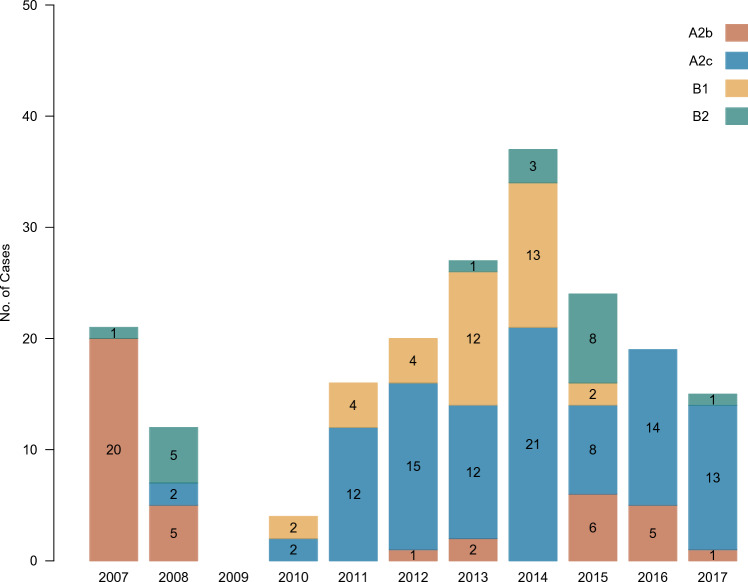
Temporal distribution of cases according to hMPV genotype and sublineage. The yearly number of cases stratified by genotype and sublineage obtained via ARI surveillance in central Vietnam during 2007–2017 is shown. Colors differentiate the genotypes and sublineages.

Among 278 hMPV-positive cases, the partial F gene was successfully amplified in 195 (70.1%) and 101 had unique sequences, defined as sequences that were not found to be identical each other. Sequences were not successfully readable in the remaining cases which may be due to low viral copies. The time-scaled phylogenetic tree showed that detected hMPV were classified into known genotypes and sublineages (Fig. 3). Of all obtained sequences, 139 (71.3%) cases were classified into subgroup A and 56 (28.7%) were classified into B. Of these, A2c predominated (n = 99), followed by A2b (n = 40), B1 (n = 37), and B2 (n = 19). A2b predominated before 2009 whereas A2c and B1 began to be detected after 2009 (Fig. 4). A2b continued to be detected even after 2012, although it was not a predominant sublineage. A2a was not identified in this study. Overall, the results showed that alternating hMPV sublineages circulated continuously over the 10-year study period.

### Molecular evolutionary characteristics of hMPV fusion (F) glycoprotein

We calculated the pairwise distances to obtain nucleotide identity between the sequences. The median nucleotide identity between all hMPV sequences was 94.0%. The median nucleotide identity of 95.2% in subgroup B was lower than that in subgroup A (98.2%), indicating that circulating hMPV subgroup B was more diverse than subgroup A. The median nucleotide identities were high in each genotype and sublineage (99.1% in A2b, 98.6% in A2c, 99.1% in B1, and 99.1% in B2).

We estimated the molecular evolutionary rate of the hMPV F gene using the Bayesian Markov Chain Monte Carlo (MCMC) method. The evolutionary rate of the F gene of hMPV subgroup A and B was estimated to be 1.2 × 10^−3^ substitutions/site/year (95% highest posterior density [HPD]: 8.9 × 10^−4^ to 1.5 × 10^−3^).

### Clinical signs and symptoms in hMPV-positive patients

Next, we described the clinical characteristics of patients with hMPV (Supplementary Table 1). Of 278 hMPV-positive children, the median age was 21.0 months (interquartile range [IQR] 12.7–32.5, and 45.3% of the children were female. Among them, 72.3% experienced tachypnea, 54.0% developed wheezing, and 29.1% developed clinical pneumonia. Other respiratory viruses were detected in 77 children (27.7%). The most frequently co-detected virus was rhinovirus/enterovirus (n = 49), followed by adenovirus (n = 10). Compared with patients without co-detected viral infection, co-infected patients were significantly younger (*p* = 0.044) and 71.4% were male children (Supplementary Table 2). The duration of hospitalization was longer in patients without viral coinfection (*p* = 0.022); however, their clinical features were not significantly different (*p* > 0.05).

To ascertain differences in the clinical features associated with detected viruses, we stratified patients into four groups: hMPV-single positive (n = 201), RSV-single positive (n = 1216), patients with RSV and hMPV (n = 5), and those with neither hMPV nor RSV (n = 6873) (Fig. 1). Patients with RSV and hMPV co-infection were not included in this analysis because of the small sample size. hMPV-single-positive children were older than RSV-single-positive children and hMPV-RSV-double-negative children (*p* < 0.001 and *p* = 0.003, respectively) (Table 2). The proportion of female patients was higher in the hMPV-single-positive group (*p* = 0.005 and *p* = 0.004, respectively). In a comparison with the hMPV-RSV-double-negative group, significantly increased risks of difficulty breathing, wheezing, crackling, and clinical pneumonia (*p* < 0.05), decreased risk of tachypnea (*p* = 0.006), and longer duration of hospitalization (*p* < 0.001) were observed in the hMPV-single-positive group (Table 2). There were no significant differences in a comparison of the hMPV-single-positive and RSV-single-positive groups in clinical characteristics; however, onset to hospitalization and onset to discharge in the hMPV-single-positive group were shorter than those in the RSV-single-positive group (*p* < 0.05).

**Table 2:** Clinical features of hMPV-single-positive patients compared with RSV-single-positive patients and hMPV-RSV-double-negative patients.

Characteristics	hMPV vs RSV			hMPV vs Non-RSV		
	hMPV, N = 201	RSV, N = 1,216	*p*-value^a^	hMPV, N = 201	Non-RSV, N = 6,873	*p*-value^b^
Age month (Median (IQR))	22.9 (12.7, 35.0)	12.9 (6.3, 22.6)	< 0.001	22.9 (12.7, 35.0)	17.9 (9.6, 30.1)	0.003
Age group (n (%))			< 0.001			< 0.001
1–5 month	11 (5.5%)	17 (1.4%)		11 (5.5%)	578 (8.4%)	
6–11 month	20 (10.0%)	286 (23.5%)		20 (10.0%)	926 (13.5%)	
12–23 month	56 (27.9%)	383 (31.5%)		56 (27.9%)	2,252 (32.8%)	
24–35 month	53 (26.4%)	175 (14.4%)		53 (26.4%)	1,111 (16.2%)	
36–47 month	19 (9.5%)	57 (4.7%)		19 (9.5%)	485 (7.1%)	
48–59 month	14 (7.0%)	13 (1.1%)		14 (7.0%)	242 (3.5%)	
≧ 60 month	28 (13.9%)	285 (23.4%)		28 (13.9%)	1,279 (18.6%)	
Sex (n (%))			0.005			0.004
Male	97 (48.3%)	716 (58.9%)		97 (48.3%)	4,023 (58.5%)	
Female	104 (51.7%)	500 (41.1%)		104 (51.7%)	2,850 (41.5%)	
Cough (n (%))	200 (99.5%)	1,213 (99.8%)	0.5	200 (99.5%)	6,847 (99.6%)	0.5
Difficulty of breathing (n (%))	68 (33.8%)	421 (34.6%)	0.8	68 (33.8%)	1,729 (25.2%)	0.005
Tachypnea (n (%))	150 (74.6%)	955 (78.5%)	0.2	150 (74.6%)	5,653 (82.2%)	0.006
Chest indrawing (n (%))	11 (5.5%)	100 (8.2%)	0.2	11 (5.5%)	299 (4.4%)	0.4
Stridor (n (%))	3 (1.5%)	12 (1.0%)	0.5	3 (1.5%)	111 (1.6%)	> 0.9
Wheeze (n (%))	105 (52.2%)	687 (56.5%)	0.3	105 (52.2%)	3,017 (43.9%)	0.019
Crackle (n (%))	38 (18.9%)	231 (19.0%)	> 0.9	38 (18.9%)	785 (11.4%)	0.001
Clinical pneumonia (n (%))			0.6			0.010
No pneumonia	148 (73.6%)	915 (75.2%)		148 (73.6%)	5,559 (80.9%)	
Pneumonia	53 (26.4%)	301 (24.8%)		53 (26.4%)	1,314 (19.1%)	
Presence of danger sign (n (%))	5 (2.5%)	37 (3.0%)	0.7	5 (2.5%)	162 (2.4%)	0.8
Body temperature (mean (SD))	38.0 (0.9)	38.0 (0.8)	0.5	38.0 (0.9)	38.1 (0.9)	0.8
Duration of hospitalization (days, median (IQR))	5.0 (4.0, 6.0)	5.0 (4.0, 6.0)	0.8	5.0 (4.0, 6.0)	4.0 (3.0, 6.0)	< 0.001
Onset to hospitalization (days, Median (IQR))	2.0 (1.0, 3.0)	3.0 (2.0, 4.0)	0.016	2.0 (1.0, 3.0)	2.0 (1.0, 4.0)	0.7
Unknown	33	151		33	806	
Onset to discharge (days, median (IQR))	7.0 (6.0, 9.0)	8.0 (6.0, 10.0)	0.015	7.0 (6.0, 9.0)	7.0 (5.0, 10.0)	0.2
Unknown	33	151		33	806	

*IQR* interquartile range, *hMPV* human metapneumovirus-single-positive group, *RSV* respiratory syncytial virus-single-positive group, *Non-RSV* human metapneumovirus–respiratory syncytial virus double-negative group.

^a^Kruskal–Wallis rank sum test; Fisher’s Exact Test for Count Data with simulated *p*-value (based on 2000 replicates); Pearson’s Chi-squared test; Fisher’s exact test; Wilcoxon rank sum test.

^b^Kruskal–Wallis rank sum test; Pearson’s Chi-squared test; Fisher’s exact test; Wilcoxon rank sum test.

Lastly, to detect any differences between hMPV cases with the various subgroups, genotypes, and sublineages, we compared demographic and clinical characteristics between patients with hMPV (Table 3). Excluding co-detected cases and those whose hMPV genotype could not be determined, we summarized the remaining 145 hMPV cases. The median patient age (in months) was not significantly different according to genotype and sublineage. The frequency of developing wheezing was significantly higher in cases with A2b (76.7%) compared with those with other genotypes and sublineages (48.7%, 42.3%, and 46.2% for A2c, B1, and B2, respectively; *p* = 0.033). To clarify the relationship between the developing wheezing to age, we conducted multiple logistic regression analysis with age adjustment. As a result, the adjusted odds ratio of developing wheezing was significantly higher in cases with A2b (adjusted odds ratio = 4.48, 95% confidence interval: 1.46, 14.9, *p* = 0.011) than cases with A2c, B1, or B2 (Supplementary Table 3). Additionally, the duration from onset to hospitalization in patients with A2b was significantly longer than the duration in patients with other genotypes and sublineages (*p* = 0.039), although more than half (18/30) of the onset dates in patients with A2b were unavailable. There was no significant difference regarding the presence of other clinical features among the subgroups, genotypes, and sublineages.

**Table 3:** Clinical features of patients with hMPV stratified by subgroups, genotypes, and sublineages.

Characteristics	A versus B			A2 versus B1 versus B2				A2b versus A2c versus B1 vs B2				
	A, N = 106	B, N = 39	*p*-value^a^	A2, N = 106	B1, N = 26	B2, N = 13	*p*-value^b^	A2b, N = 30	A2c, N = 76	B1, N = 26	B2, N = 13	*p*-value^b^
Age Month (median (IQR))	24.0 (13.5, 31.3)	23.7 (8.9, 35.3)	0.8	24.0 (13.5, 31.3)	23.2 (8.8, 35.5)	25.0 (10.0, 34.6)	> 0.9	23.9 (15.6, 31.3)	24.0 (13.4, 30.9)	23.2 (8.8, 35.5)	25.0 (10.0, 34.6)	> 0.9
Age group (n (%))			0.13				0.3					0.5
1–5 month	3 (2.8%)	0 (0.0%)		3 (2.8%)	0 (0.0%)	0 (0.0%)		0 (0.0%)	3 (3.9%)	0 (0.0%)	0 (0.0%)	
6–11 month	10 (9.4%)	4 (10.3%)		10 (9.4%)	4 (15.4%)	0 (0.0%)		2 (6.7%)	8 (10.5%)	4 (15.4%)	0 (0.0%)	
12–23 month	31 (29.2%)	7 (17.9%)		31 (29.2%)	5 (19.2%)	2 (15.4%)		9 (30.0%)	22 (28.9%)	5 (19.2%)	2 (15.4%)	
24–35 month	35 (33.0%)	11 (28.2%)		35 (33.0%)	6 (23.1%)	5 (38.5%)		11 (36.7%)	24 (31.6%)	6 (23.1%)	5 (38.5%)	
36–47 month	7 (6.6%)	7 (17.9%)		7 (6.6%)	5 (19.2%)	2 (15.4%)		3 (10.0%)	4 (5.3%)	5 (19.2%)	2 (15.4%)	
48–59 mo	8 (7.5%)	1 (2.6%)		8 (7.5%)	1 (3.8%)	0 (0.0%)		1 (3.3%)	7 (9.2%)	1 (3.8%)	0 (0.0%)	
≧ 60 month	12 (11.3%)	9 (23.1%)		12 (11.3%)	5 (19.2%)	4 (30.8%)		4 (13.3%)	8 (10.5%)	5 (19.2%)	4 (30.8%)	
Sex (n (%))			0.12				0.3					0.5
Male	56 (52.8%)	15 (38.5%)		56 (52.8%)	10 (38.5%)	5 (38.5%)		16 (53.3%)	40 (52.6%)	10 (38.5%)	5 (38.5%)	
Female	50 (47.2%)	24 (61.5%)		50 (47.2%)	16 (61.5%)	8 (61.5%)		14 (46.7%)	36 (47.4%)	16 (61.5%)	8 (61.5%)	
Cough (n (%))	105 (99.1%)	39 (100.0%)	> 0.9	105 (99.1%)	26 (100.0%)	13 (100.0%)	> 0.9	30 (100.0%)	75 (98.7%)	26 (100.0%)	13 (100.0%)	> 0.9
Difficulty of breathing (n (%))	39 (36.8%)	14 (35.9%)	> 0.9	39 (36.8%)	11 (42.3%)	3 (23.1%)	0.5	11 (36.7%)	28 (36.8%)	11 (42.3%)	3 (23.1%)	0.7
Tachypnea (n (%))	78 (73.6%)	28 (71.8%)	0.8	78 (73.6%)	17 (65.4%)	11 (84.6%)	0.4	21 (70.0%)	57 (75.0%)	17 (65.4%)	11 (84.6%)	0.6
Chest indrawing (n (%))	7 (6.6%)	2 (5.1%)	> 0.9	7 (6.6%)	2 (7.7%)	0 (0.0%)	0.9	2 (6.7%)	5 (6.6%)	2 (7.7%)	0 (0.0%)	> 0.9
Stridor (n (%))	1 (0.9%)	0 (0.0%)	> 0.9	1 (0.9%)	0 (0.0%)	0 (0.0%)	> 0.9	0 (0.0%)	1 (1.3%)	0 (0.0%)	0 (0.0%)	> 0.9
Wheeze (n (%))	60 (56.6%)	17 (43.6%)	0.2	60 (56.6%)	11 (42.3%)	6 (46.2%)	0.4	23 (76.7%)	37 (48.7%)	11 (42.3%)	6 (46.2%)	0.033
Crackle (n (%))	20 (18.9%)	8 (20.5%)	0.8	20 (18.9%)	5 (19.2%)	3 (23.1%)	> 0.9	7 (23.3%)	13 (17.1%)	5 (19.2%)	3 (23.1%)	0.8
Clinical pneumonia (n (%))			> 0.9				0.5					0.7
No pneumonia	76 (71.7%)	28 (71.8%)		76 (71.7%)	17 (65.4%)	11 (84.6%)		21 (70.0%)	55 (72.4%)	17 (65.4%)	11 (84.6%)	
Pneumonia	30 (28.3%)	11 (28.2%)		30 (28.3%)	9 (34.6%)	2 (15.4%)		9 (30.0%)	21 (27.6%)	9 (34.6%)	2 (15.4%)	
Presence of danger sign (n (%))	1 (0.9%)	2 (5.1%)	0.2	1 (0.9%)	2 (7.7%)	0 (0.0%)	0.10	0 (0.0%)	1 (1.3%)	2 (7.7%)	0 (0.0%)	0.2
Body temperature (mean (SD))	38.0 (0.9)	38.1 (0.7)	0.4	38.0 (0.9)	38.0 (0.7)	38.3 (0.7)	0.3	38.1 (0.9)	38.0 (0.9)	38.0 (0.7)	38.3 (0.7)	0.5
Respiratory rate (per min, mean (SD))	36 (9)	37 (10)	> 0.9	36 (9)	39 (11)	34 (6)	0.4	36 (11)	37 (9)	39 (11)	34 (6)	0.4
Duration of hospitalization (days, median (IQR))	5.0 (4.0, 6.0)	6.0 (4.0, 7.0)	0.15	5.0 (4.0, 6.0)	5.0 (4.0, 6.0)	6.0 (5.0, 7.0)	0.2	5.0 (4.0, 6.0)	5.0 (3.0, 6.0)	5.0 (4.0, 6.0)	6.0 (5.0, 7.0)	0.4
Onset to hospitalization (days, median (IQR))	2.5 (2.0, 4.0)	2.0 (1.0, 3.0)	0.2	2.5 (2.0, 4.0)	2.0 (1.0, 3.0)	2.0 (1.0, 3.0)	0.4	4.0 (2.8, 5.0)	2.0 (1.8, 3.0)	2.0 (1.0, 3.0)	2.0 (1.0, 3.0)	0.039
Unknown	18	3		18	0	3		18	0	0	3	
Onset to discharge (days, median (IQR))	8.0 (6.0, 9.0)	8.0 (6.0, 9.0)	0.8	8.0 (6.0, 9.0)	7.5 (6.0, 9.0)	8.0 (7.0, 8.8)	0.9	8.5 (7.0, 10.2)	8.0 (5.0, 9.0)	7.5 (6.0, 9.0)	8.0 (7.0, 8.8)	0.5
Unknown	18	3		18	0	3		18	0	0	3	

*hMPV* human metapneumovirus, *IQR* interquartile range, *SD* standard deviation.

^a^Kruskal–Wallis rank sum test; Fisher’s Exact Test for Count Data with simulated *p*-value (based on 2000 replicates); Pearson’s Chi-squared test; Fisher’s exact test; Wilcoxon rank sum test,

^b^Kruskal–Wallis rank sum test; Fisher’s Exact Test for Count Data with simulated *p*-value (based on 2000 replicates); Pearson’s Chi-squared test; Fisher’s exact test.

Two patients were admitted twice for hMPV infection during the study period. One of them was infected with B1 in 2010 and with A2c in 2012. The F gene sequences were undetermined in the other patient.

## Discussion

We conducted ARI surveillance among hospitalized children in central Vietnam over 10 years and detected hMPV together with other common respiratory viruses. This study revealed the continuous circulation of hMPV, the predominant hMPV genotype, the genetic diversity, and clinical manifestations in pediatric ARI patients with hMPV. Specifically, we focused on the evolutionary characteristics of the hMPV F gene to help inform hMPV vaccine development and monoclonal antibodies discovery.

A global comparative review revealed that 80% of tropical locations experience distinct RSV seasons^20^. We also observed apparent seasonality of RSV whereas hMPV circulated without apparent seasonal forcing. Previous studies have reported that the seasonality of hMPV outbreaks varies among tropical and Asian countries. In Kenya^21^, occurrences of hMPV correspond to the season with lower rainfall and high temperatures. In Senegal^22^, clear seasonality related to the rainy season has been observed. In South Korea^23^ and Japan^24^, seasonality has been found from winter to spring, and in China^25^, seasonality was observed from spring to early summer. However, no clear seasonality of hMPV has been found in Bangladesh^26^, or Cambodia^27^. Although previous research has suggested a correlation between the prevalence of hMPV and meteorological factors such as high relative humidity and rainy days in Malaysia^28^, the dynamics of hMPV in tropical regions are not yet well understood. Differences in the circulating genotype and surveillance methods among countries and regions may affect study findings; therefore, comprehensive data collection and meta-analysis are necessary.

We only identified five hMPV-positive cases in 2009–2010. Regarding this period, we reported that influenza A and B activities were also suppressed from December 2009 to February 2010 after the start of the influenza A H1N1 pandemic 2009 at the study site in July 2009^29^. Additionally, a German study reported that the 2009/2010 hMPV epidemic started with a delay; the authors believed that this was possibly because hMPV and influenza A H1N1/pdm2009 virus were competing for human hosts^30^. Moreover, a recent finding showed that social distancing measures implemented for coronavirus disease 2019 might be associated with reductions in common respiratory viruses^31^. Taking these observations into account, we speculate that human behavioral changes, such as reducing close contacts, may have contributed to the decreased activities of hMPV as well as RSV during the influenza A H1N1 pandemic 2009.

In our study, hMPV subgroups were detected simultaneously, with the predominant circulating genotype and sublineages consistently alternating during the study period. Specifically, A2b was predominately seen before 2009, whereas A2c predominated in and after 2011. Notably, A2a was not observed through the study period in our study. However, the study by Yi et al.^25^, which summarized the continental distribution of hMPV genotypes, indicated that A2a continued to be observed in America from 2006 to 2017^25^.

It is worth noting that A2c may have been introduced into the study area and became predominant despite the A2b was continued to be detected. Concurrent circulation of multiple genotypes and sublineages is a common feature in other parts of the world where hMPV genetic diversity has been monitored for five or more years^21,25,32–34^. It is unclear whether A2c has antigenicity that differs enough to escape A2b-specific immunity. Small animal models have shown a high degree of cross-neutralization between genotypes but a low degree of cross-neutralization between subgroups^19^. Taken together, A2c became predominant over A2b because it might be advantageous to its circulation in respect of transmissibility or proliferation. Further serological and virological analyses are necessary to clarify this point.

A high nucleotide identity of hMPV F gene sequences was found in our study, which is in agreement with another study spanning 20 years^33^. Both overall and in several sites were reported to be under negative (i.e., purifying) selection in the F gene^32^. Our estimated evolutionary rate of the F gene was comparable to those of previous estimations for the F gene of human and avian metapneumovirus: (6.0 × 10^−4^ to 1.3 × 10^−3^substitution/site/year)^35^, although viral substitution rates can be underestimated under purifying selection^36^. A larger genetic diversity^18^ and a faster evolutionary rate (3.2–5.4 × 10^−3^ substitution/site/year) in the G gene than in the F gene have been reported^37^. This difference may arise from the roles of these coding proteins in response to the host immune system. A previous study hypothesized that the extracellular ectodomain of the G protein sterically masks the pre-fusion hMPV-F from the host immune response, which may explain why infection only confers transient immunity resulting in reinfection despite relatively low evolutionary rates in the F gene of hMPV^38^. A recent study characterizing antibodies specific for the hMPV F protein identified neutralizing antibodies that recognize pre-fusion-specific epitopes in adult donors, and these provided robust protection against lower respiratory infection in a small animal model^39^. Hence, the F gene may offer a useful target for monoclonal antibodies or vaccines in terms of its genetic stability and antigenicity.

We illustrated that clinical manifestations of hMPV patients are indistinguishable from those with RSV and those with co-infection of other viruses, which is consistent with a previous study^8^. We found that 52% of patients in the hMPV-single-positive group showed wheezing, which had good concordance with a previous large study reporting a proportion of 52%^40^. We also found an increased frequency of developing wheezing among cases with A2b compared with A2c, B1, and B2 cases. An association between genotype and clinical features remains elusive and various insights have been obtained in previous studies. Infection with subgroup A was associated with severe disease in a Spanish study^41^, and infection with B1 or B2 was associated with an increased risk of wheezing compared with A2 in a Japanese study^5^. Whereas, hMPV viral loads, which have been reported to be associated with the disease severity^42^, estimated by cycle threshold values were not different between those with detected genotypes nor subgroups in a Nepalese study^21^. A hospital study in Taiwan also did not find any difference in clinical features among genotypes or subgroups^43^. The variations may be influenced by the occurrence of sublineages, previous outbreaks, the generally limited sample sizes in hospital-based surveillance, and the timing when clinical features were evaluated in the course of the disease. Although we found a few cases of hMPV reinfection in this study, the frequency of reinfections may be also associated with the variation. These points should be clarified in studies among an hMPV-naive population, such as a birth cohort, and to identify the underlying mechanism causing the variation.

This study had some limitations. First, we did not examine bacterial infection. An increased hMPV seroconversion rate was reported to be associated with a greater frequency of *S. pneumoniae* colonization in epidemiological study^44^. The study also demonstrated that the prior exposure to *S. pneumoniae* was found to be associated with greater susceptibility of cell to hMPV infection in vitro experiments. We believe it is unlikely that the co-infecting *S. pneumoniae* affects the hMPV genotype-specific clinical features. However, the circulation level of *S. pneumoniae* may have temporarily influenced the hMPV incidence. Future studies will clarify this point when influences of *S. pneumoniae* on the hMPV disease are available. Second, a general caveat of molecular epidemiology studies is applicable, namely, the possibility of a false negative test result in samples with an undetected variant. To clarify the effect on our results, hMPV-negative samples tested using the present method should be tested using whole-genome sequencing to identify any undetected variants. A study that conducted a metagenomic sequencing analysis of 190 samples that were negative in a standard virus diagnostic panel revealed that 3.2% of the samples were hMPV positive but the positive results had been missed^45^. We used a genetically stable hMPV gene to screen samples; thus, we believe the likelihood of missed positive samples is minimal. Although we could not clarify this point due to the limited availability of clinical specimen volume, further investigation can provide greater insight into hMPV genomic diversity and its incidence in central Vietnam. Third, only NPS specimens were collected in this study. However, a previous study recommended bronchoalveolar lavage fluid (BALF) for diagnosing lower respiratory infections, especially in severe cases^46^. Since no patient was severe enough to receive invasive respiratory support in this study, BALF specimens were not collected. Further analyses of severe cases using BALF specimens may allow us to identify causative agents of lower respiratory infections more accurately and specifically.

Despite these limitations, we can conclude that hMPV infection significantly affects public health among hospitalized children with ARI in central Vietnam, as it can cause severe illness that is comparable in severity to RSV, despite affecting older age groups. We found the year-round continuous occurrence of hMPV infection and limited genetic diversity of the F gene in long-term monitoring, along with moderate variation in the clinical features of patients with hMPV infection. Future interventional studies that include the introduction of vaccines or monoclonal antibodies will help to ease the impact of hMPV-associated infection on public health.

## Methods

### Study site, study period, and participant enrollment

We initiated a pediatric ARI surveillance at Khanh Hoa Provincial General Hospital (KHGH) in Nha Trang, Vietnam beginning in February 2007^47^. Briefly, children aged 1 month to 14 years who were residing in 16 communes in Nha Trang, admitted to the KHGH pediatric ward presenting ARI symptoms from February 2007 through December 2017 were invited to participate in the current study. The study area covered approximately 20,037 and 20,174 children under five in 2009 and 2015, respectively (personal communication with Dr. Minh Xuan Bui, Khanh Hoa Health Service). ARI symptoms were defined as the presence of cough or difficulty breathing. The presence of difficulty in breathing was determined through pediatrician observations when the child exhibited abnormal respiratory patterns such as noisy, interrupted, or irregular respiratory rates. Before the study enrollment, written informed consent was obtained from the legal guardians of all children. The research protocol to run the study was approved by the institutional ethical review boards of both the National Institute of Hygiene and Epidemiology, Vietnam (IRB-VN 01057) and the Institute of Tropical Medicine, Nagasaki University, Japan (09031837-3). The study was conducted in accordance with approved guidelines and regulations.

### Data collection

Upon study enrollment, nasopharyngeal swab (NPS) samples and clinical and epidemiological information were collected. For clinical categorization, we used the definition of the modified World Health Organization Integrated Management of Childhood Illnesses algorithm, in which the presence of tachypnea (respiratory rate ≥ 60/min for children aged < 2 months, ≥ 50/min for ages 2–11 months, ≥ 40/min for ages 1–5 years, ≥ 30/min for ages 6–11 years, and ≥ 20/min for children aged 12 years or older) or chest indrawing was categorized as clinical pneumonia^48,49^. We re-defined the presence of difficulty in breathing in our analysis to include the presence of tachypnea or chest indrawing. Furthermore, children with general danger signs (situations in which the child is either unable to drink or has altered consciousness, convulsions, lethargy, poor sucking, toxic appearance, or irritability) were also recorded.

### Respiratory virus screening using multiplex PCR and hMPV F gene sequencing

Viral nucleic acids were extracted from NPS samples using a QIAamp viral RNA Mini kit (QIAGEN Inc., Valencia, CA, USA). Four sets of multiplex PCR assays were used for screening 13 respiratory viruses, separately testing for RSV, hMPV, influenza virus A, influenza virus B, parainfluenza viruses (types 1, 2, 3, and 4), rhinovirus/enterovirus, human coronaviruses (229E and OC43), adenovirus, and bocavirus^47^. All positive specimens for each virus were confirmed with hemi-nested PCR^47^. For hMPV-positive samples, we amplified the partial F gene by PCR and performed sequencing reactions. Briefly, we amplified the gene coding partial F protein (527 base pairs) of hMPV-positive samples using primers as previously described^50^, and performed sequencing reactions with a BigDye Terminator v1.1 or v3.1 cycle sequencing kit (Applied Biosystems, Foster City, CA, USA) after purification of the PCR product using ExoSAP-IT Express (Thermo Fisher Scientific, Waltham, MA, USA). We conducted nucleotide sequence analysis using either a 3130 or 3730XL DNA Analyzer (Applied Biosystems).

### Phylogenetic and molecular evolutionary analysis

To characterize the influence of viral genomic diversity on changes in the circulation patterns and clinical manifestations of hMPV, we conducted phylogenetic and molecular evolutionary analysis for obtained F gene sequences. HMPV subgroups A and B were further classified into genotypes A1, A2, B1, and B2 based on genetic differences^18^. A2 can be further categorized into several branches, A2a, A2b, and A2c, according to F gene phylogenetic analysis^51^. We, therefore, differentiated hMPV A2a, A2b, and A2c as sublineages of genotype A2 in our analysis. To classify the obtained sequences into these genotypes and sublineages, we conducted a phylogenetic analysis with reference sequences. The accession numbers of representative reference sequences used for the analysis were described in Supplementary Material.

In addition, we estimated the evolutionary rate of the partial F gene. Briefly, the time-scaled phylogenetic tree was inferred using Bayesian Markov Chain Monte Carlo (MCMC) with BEAST software version 2.6.6 under a coalescent constant population on the tree and strict clock model^52^. The best fit substitution model was selected using bModeltest implemented in BEAST software^53^. The MCMC chains were run for sufficient steps to achieve convergence. Tracer version 1.7.2 was used to assess the convergence based on effective sample size (ESS) after 10% burn-in; parameters with ESS greater than 200 were accepted. The time-scaled maximum clade credibility (MCC) tree was generated with Tree Annotator version 1.8.3 after excluding the first 10% of trees as a burn-in, and was viewed with R *ggtree* package^54^. The nucleotide identity was also calculated based on pairwise distances between each sequence to evaluate the genetic variation within hMPV subgroups, genotypes and sublineages using R *ape* package^55^. The hMPV sequences with 100% nucleotide identity were intentionally excluded from these analyses.

### Clinical data analysis

We recorded information on the children’s age, sex, and clinical symptoms, including cough, difficulty breathing, tachypnea, chest indrawing, stridor, wheezing, crackle, clinical pneumonia, presence of danger signs, body temperature, and respiratory rate. To determine those characteristics that are attributable to the detected virus, we stratified patients into four groups according to the results of viral screening: patients with hMPV infection only (hMPV-single-positive group), patients with RSV infection only (RSV-single-positive group), patients with both hMPV and RSV infections (hMPV-RSV-double-positive group), and patients with neither hMPV nor RSV (hMPV-RSV-double-negative group). Detailed information on RSV patients in this surveillance in 2010–2012 was previously reported^56^. We compared the clinical characteristics of the hMPV-single-positive group to those with hMPV and viral coinfection, including the hMPV-RSV-double-positive group, however, those were not included in further analysis. The characteristics of hMPV-single-positive patients were compared with those of RSV-single-positive patients and those of hMPV-RSV-double-negative patients using simple statistical tests. We then compared the characteristics of hMPV according to subgroup, genotype, and sublineage using simple statistical tests. To further investigate the relationship between age and variables associated with subgroup, genotype, and sublineage, we conducted multiple logistic regression analysis with age adjustment. Moreover, we described patients who were repeatedly hospitalized for hMPV infection during the study period.

For comparisons of categorical variables between two or three independent groups, we used either the two-tailed Pearson’s chi-squared or Fisher’s exact test. For continuous variables, we used the Wilcoxon rank sum test for comparisons between two independent groups and the Kruskal–Wallis test for comparisons among three or more independent groups. All statistical analyses were performed with R version 4.1.2^57^. A *p*-value less than 0.05 was taken as statistically significant.

### Supplementary Information

Supplementary Information.

## Data Availability

The nucleotide sequences for each genotype and sublineage have been registered in GenBank. The accession numbers in GenBank are OP947591–OP947594.
