# Supplementary material for: A decade of human metapneumovirus in hospitalized children with acute respiratory infection: molecular epidemiology in central Vietnam, 2007–2017
Source: Sci Rep. 2023 Sep 21;13:15757. doi: 10.1038/s41598-023-42692-z (PMC10514255; doi:10.1038/s41598-023-42692-z)
Supplement: Supplementary file 1 — Supplementary Information. [file 41598_2023_42692_MOESM1_ESM.pdf]

Supplementary Materials

Article Title: A decade of human metapneumovirus in hospitalized children with acute respiratory infection: Molecular epidemiology in central Vietnam, 2007-2017

Authors and Affiliations:
Hirono Otomaru[1], Hien Anh Thi Nguyen[2], Hien Minh Vo[3], Michiko Toizumi[1], Minh Nhat Le[4,5], Katsumi Mizuta[6], Hiroyuki Moriuchi[7], Minh Xuan Bui[8], Duc Anh Dang[9], and Lay-Myint Yoshida[1,10]

1. Department of Pediatric Infectious Diseases, Institute of Tropical Medicine, Nagasaki University, Nagasaki, Japan
2. Department of Bacteriology, National Institute of Hygiene and Epidemiology, Hanoi, Vietnam
3. Department of Pediatrics, Khanh Hoa General Hospital, Nha Trang, Vietnam
4. Antimicrobial Resistance Research Centre, National Institute of Infectious Disease (NIID), Shinjuku, Japan
5. Tay Nguyen Institute of Science Research, Vietnam Academy of Science and Technology (VAST), Da Lat, Vietnam
6. Yamagata Prefectural Institute of Public Health, Yamagata, Japan
7. Department of Pediatrics, Nagasaki University Graduate School of Biomedical Sciences, Nagasaki, Japan
8. Khanh Hoa Health Service Department, Nha Trang, Vietnam
9. National Institute of Hygiene and Epidemiology, Hanoi, Vietnam
10. Graduate School of Biomedical Sciences, Nagasaki University, Nagasaki, Japan

Subgroup, genotype, and sublineage classification of hMPV

HMPV subgroup A and B were further classified into genotypes A1, A2, B1, and B2 based on genetic differences in a previous report [1]. A recent study reported that hMPV A2 can be further categorized into several branches, A2a, A2b, and A2c, according to F gene phylogenetic analysis [2]. Furthermore, variants with 180- or 111-nucleotide duplication in the G gene were found to be included in the A2c branch in phylogenetic analysis of the F and G gene [3–5]. We therefore differentiated hMPV A2a, A2b, and A2c as sublineages of genotype A2 in our analysis. To classify the obtained sequences into these genotypes and sublineages, we conducted a phylogenetic analysis with reference sequences. The hMPV F gene sequences were aligned with the Clustal W algorithm implemented within *msa* package [6] in R version 4.1.2 [7]. Accession numbers in GeneBank of the representative reference sequences used for classification were AF371337 (A1), AB503857 (A2a), AY530095 (A2b), GQ153651 (A2c), AY525843 (B1), and FJ168778 (B2) [8]. Accession numbers in GeneBank of our sequences for representatives in genotypes and sublineages are OP947591–OP947594. Samples that failed to be amplified were classified as genotype undetermined.

References

1. Ishiguro N, Ebihara T, Endo R, et al. High genetic diversity of the attachment (G) protein of human metapneumovirus. J Clin Microbiol. **2004**; 42(8):3406–3414.
2. Nidaira M, Taira K, Hamabata H, et al. Molecular epidemiology of human metapneumovirus from 2009 to 2011 in Okinawa, Japan. Jpn J Infect Dis. **2012**; 65(4):337–340.
3. Piñana M, Vila J, Gimferrer L, et al. Novel human metapneumovirus with a 180-nucleotide duplication in the G gene. Future Microbiol. **2017**; 12(7):565–571.
4. Saikusa M, Nao N, Kawakami C, et al. A novel 111-nucleotide duplication in the G gene of human metapneumovirus. Microbiol Immunol. **2017**; 61(11):507–512.
5. Piñana M, Vila J, Maldonado C, et al. Insights into immune evasion of human metapneumovirus: novel 180- and 111-nucleotide duplications within viral G gene throughout 2014-2017 seasons in Barcelona, Spain. J Clin Virol. **2020**; 132:104590.
6. Bodenhofer U, Bonatesta E, Horejš-Kainrath C, Hochreiter S. msa: an R package for multiple sequence alignment. Bioinformatics. **2015**; 31(24):3997–3999.
7. R Core Team. R: A language and environment for statistical computing [Internet]. Vienna, Austria: R Foundation for Statistical Computing; 2021. Available from: https://www.R-project.org/
8. Oketch JW, Kamau E, Otieno GP, Otieno JR, Agoti CN, Nokes DJ. Human metapneumovirus prevalence and patterns of subgroup persistence identified through surveillance of pediatric pneumonia hospital admissions in coastal Kenya, 2007–2016. BMC Infect Dis. **2019**; 19(1):757.

Supplementary Table 1. Clinical features of patients with hMPV

Supplementary Table 1						
	**hMPV vs RSV**			**hMPV vs Non-RSV**		
**Characteristics**	**hMPV**, N = 278	**RSV**, N = 1,671	**p-value**[a]	**hMPV**, N = 278	**Non-RSV**, N = 6,873	**p-value**[b]
**Age Month (Median (IQR))**	21.0 (12.7, 32.5)	13.9 (6.8, 22.6)	<0.001	21.0 (12.7, 32.5)	17.9 (9.6, 30.1)	0.011
**Age Group (n (%))**			<0.001			<0.001
1-5mo	13 (4.7%)	24 (1.4%)		13 (4.7%)	578 (8.4%)	
6-11mo	29 (10.4%)	363 (21.7%)		29 (10.4%)	926 (13.5%)	
12-23mo	93 (33.5%)	577 (34.5%)		93 (33.5%)	2,252 (32.8%)	
24-35mo	69 (24.8%)	252 (15.1%)		69 (24.8%)	1,111 (16.2%)	
36-47mo	22 (7.9%)	70 (4.2%)		22 (7.9%)	485 (7.1%)	
48-59mo	16 (5.8%)	18 (1.1%)		16 (5.8%)	242 (3.5%)	
≧60mo	36 (12.9%)	367 (22.0%)		36 (12.9%)	1,279 (18.6%)	
**Sex (n (%))**			0.2			0.2
Male	152 (54.7%)	980 (58.6%)		152 (54.7%)	4,023 (58.5%)	
Female	126 (45.3%)	691 (41.4%)		126 (45.3%)	2,850 (41.5%)	
**Cough (n (%))**	277 (99.6%)	1,667 (99.8%)	0.5	277 (99.6%)	6,847 (99.6%)	>0.9
**Difficulty of Breathing (n (%))**	98 (35.3%)	579 (34.6%)	0.8	98 (35.3%)	1,729 (25.2%)	<0.001
**Tachypnea (n (%))**	201 (72.3%)	1,314 (78.6%)	0.019	201 (72.3%)	5,653 (82.2%)	<0.001
**Chest Indrawing (n (%))**	18 (6.5%)	134 (8.0%)	0.4	18 (6.5%)	299 (4.4%)	0.092
**Stridor (n (%))**	4 (1.4%)	17 (1.0%)	0.5	4 (1.4%)	111 (1.6%)	>0.9
**Wheeze (n (%))**	150 (54.0%)	935 (56.0%)	0.5	150 (54.0%)	3,017 (43.9%)	<0.001
**Crackle (n (%))**	52 (18.7%)	292 (17.5%)	0.6	52 (18.7%)	785 (11.4%)	<0.001
**Clinical Pneumonia (n (%))**			0.10			<0.001

Supplementary Table 1

Characteristics	hMPV vs RSV			hMPV vs Non-RSV		
	**hMPV**, N = 278	**RSV**, N = 1,671	**p-value**[a]	**hMPV**, N = 278	**Non-RSV**, N = 6,873	**p-value**[b]
No pneumonia	197 (70.9%)	1,261 (75.5%)		197 (70.9%)	5,559 (80.9%)	
Pneumonia	81 (29.1%)	410 (24.5%)		81 (29.1%)	1,314 (19.1%)	
**Presence of Danger Sign (n (%))**	9 (3.2%)	47 (2.8%)	0.7	9 (3.2%)	162 (2.4%)	0.3
**Body Temperature (Mean (SD))**	38.1 (0.8)	38.0 (0.8)	0.5	38.1 (0.8)	38.1 (0.9)	0.6
**Duration of Hospitalization (days, Median (IQR))**	5.0 (3.0, 6.0)	5.0 (4.0, 7.0)	0.10	5.0 (3.0, 6.0)	4.0 (3.0, 6.0)	<0.001
**Onset to Hospitalization (days, Median (IQR))**	2.0 (1.0, 4.0)	3.0 (2.0, 4.0)	0.042	2.0 (1.0, 4.0)	2.0 (1.0, 4.0)	0.8
Unknown	45	216		45	806	
**Onset to Discharge (days, Median (IQR))**	7 (5, 9)	8 (6, 10)	0.002	7.0 (5.0, 9.0)	7.0 (5.0, 10.0)	0.2
Unknown	45	216		45	806	

[a]Kruskal-Wallis rank sum test; Fisher's Exact Test for Count Data with simulated p-value (based on 2000 replicates); Pearson's Chi-squared test; Fisher's exact test; Wilcoxon rank sum test

[b]Kruskal-Wallis rank sum test; Pearson's Chi-squared test; Fisher's exact test; Wilcoxon rank sum test

Abbreviations: IQR, interquartile range; hMPV, human metapneumovirus-positive group, RSV: respiratory syncytial virus-positive group, Non-RSV: human metapneumovirus–respiratory syncytial virus double-negative group

Supplementary Table 2. Comparison between hMPV patients with or without co-detected virus

Supplementary Table 2

Characteristics	Overall, N = 278	hMPV with Co-detected Virus		
		**No**, N = 201	**Yes**, N = 77	**p-value**[a]
**Age Month (Median (IQR))**	21.0 (12.7, 32.5)	22.9 (12.7, 35.0)	18.9 (13.0, 26.8)	0.044
**Age Group (n (%))**				0.052
1-5mo	13 (4.7%)	11 (5.5%)	2 (2.6%)	
6-11mo	29 (10.4%)	20 (10.0%)	9 (11.7%)	
12-23mo	93 (33.5%)	56 (27.9%)	37 (48.1%)	
24-35mo	69 (24.8%)	53 (26.4%)	16 (20.8%)	
36-47mo	22 (7.9%)	19 (9.5%)	3 (3.9%)	
48-59mo	16 (5.8%)	14 (7.0%)	2 (2.6%)	
≧60mo	36 (12.9%)	28 (13.9%)	8 (10.4%)	
**Sex (n (%))**				<0.001
Male	152 (54.7%)	97 (48.3%)	55 (71.4%)	
Female	126 (45.3%)	104 (51.7%)	22 (28.6%)	
**Cough (n (%))**	277 (99.6%)	200 (99.5%)	77 (100.0%)	>0.9
**Difficulty of Breathing (n (%))**	98 (35.3%)	68 (33.8%)	30 (39.0%)	0.4
**Tachypnea (n (%))**	201 (72.3%)	150 (74.6%)	51 (66.2%)	0.2
**Chest Indrawing (n (%))**	18 (6.5%)	11 (5.5%)	7 (9.1%)	0.3
**Stridor (n (%))**	4 (1.4%)	3 (1.5%)	1 (1.3%)	>0.9
**Wheeze (n (%))**	150 (54.0%)	105 (52.2%)	45 (58.4%)	0.4
**Crackle (n (%))**	52 (18.7%)	38 (18.9%)	14 (18.2%)	0.9
**Clinical Pneumonia (n (%))**				0.10
No pneumonia	197 (70.9%)	148 (73.6%)	49 (63.6%)	
Pneumonia	81 (29.1%)	53 (26.4%)	28 (36.4%)	
**Presence of Danger Sign (n (%))**	9 (3.2%)	5 (2.5%)	4 (5.2%)	0.3

Supplementary Table 2

Characteristics	**Overall**, N = 278	hMPV with Co-detected Virus		
		**No**, N = 201	**Yes**, N = 77	**p-value**[a]
**Body Temperature (Mean (SD))**	38.1 (0.8)	38.0 (0.9)	38.2 (0.8)	0.2
**Duration of Hospitalization (days, Median (IQR))**	5.0 (3.0, 6.0)	5.0 (4.0, 6.0)	4.0 (3.0, 6.0)	0.022
**Onset to Hospitalization (days, Median (IQR))**	2.0 (1.0, 4.0)	2.0 (1.0, 3.0)	3.0 (1.0, 5.0)	0.2
Unknown	45	33	12	
**Onset to Discharge (days, Median (IQR))**	7.0 (5.0, 9.0)	7.0 (6.0, 9.0)	7.0 (5.0, 11.0)	0.8
Unknown	45	33	12	

[a]Kruskal-Wallis rank sum test; Fisher's Exact Test for Count Data with simulated p-value (based on 2000 replicates); Pearson's Chi-squared test; Fisher's exact test; Wilcoxon rank sum test

Abbreviations: IQR, interquartile range; hMPV, human metapneumovirus

Supplementary Table 3. Association between subgroup, genotypes, and sublineage and wheeze frequency adjusted for age months.

Supplementary Table 3															
	A vs B					A2 vs B1 vs B2					A2b vs A2c vs B1 vs B2				
**Characteristic**	**N**	**Event N**	**OR[a]**	**95% CI[a]**	**p-value**	**N**	**Event N**	**OR[a]**	**95% CI[a]**	**p-value**	**N**	**Event N**	**OR[a]**	**95% CI[a]**	**p-value**
**Age Months**	145	77	1.00	0.98, 1.02	>0.9	145	77	1.00	0.98, 1.02	>0.9	145	77	1.00	0.98, 1.02	0.9
**Subgroup**															
A	106	60	—	—											
B	39	17	0.59	0.28, 1.24	0.2										
**Genotype**															
B1						26	11	—	—						
B2						13	6	1.17	0.30, 4.52	0.8					
A2						106	60	1.78	0.75, 4.34	0.2					
**Sublineage**															
B1											26	11	—	—	
B2											13	6	1.17	0.30, 4.51	0.8
A2c											76	37	1.29	0.52, 3.23	0.6
A2b											30	23	4.48	1.46, 14.9	**0.011**

[a]OR = Odds Ratio, CI = Confidence Interval

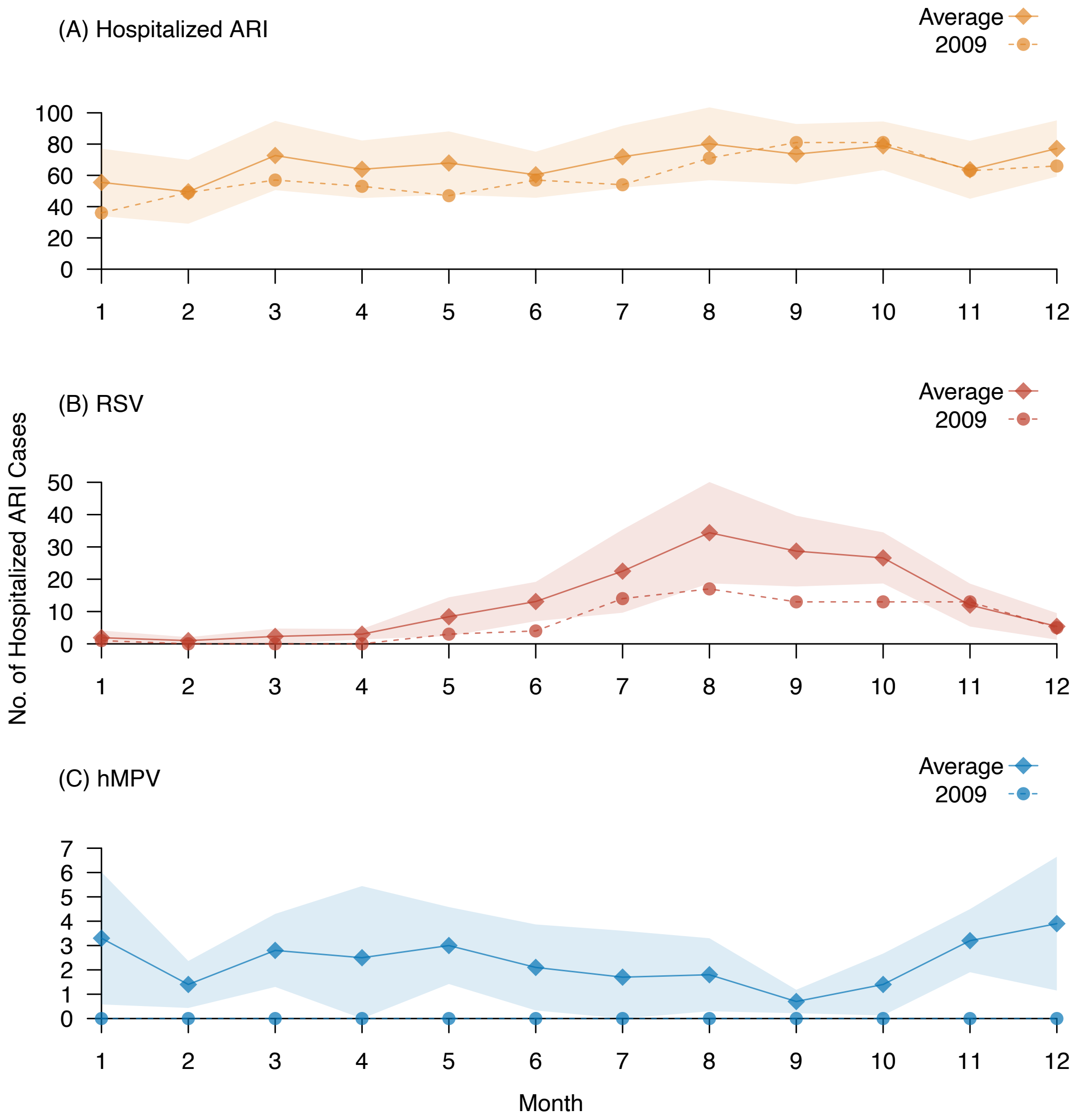

Supplementary Figure 1

The monthly number of cases for (A) hospitalized ARI, (B) RSV-positive, and (C) hMPV-positive. The solid lines with diamonds show the monthly average cases from 2007 to 2017 with the 95% confidence interval, except for 2009. The dashed lines with circles show the number of hospitalized ARI cases in 2009.
